# 

**Published:** 1892-10-15

**Authors:** 


No. 316. Vol. XIII.] Saturday, October 15th, 1892. [Twopence.

